# Fetal programming of early-onset type 2 diabetes: a Swedish nationwide cohort and sibling analysis

**DOI:** 10.1007/s10654-025-01261-6

**Published:** 2025-06-24

**Authors:** Coralie Amadou, Yuxia Wei, Tiinamaija Tuomi, Maria Feychting, Sofia Carlsson

**Affiliations:** 1 Institute of Environmental Medicine, Karolinska Institutet, Stockholm, Sweden; 2 Sud-Francilien Hospital, Paris-Saclay University, Corbeil-Essonnes, France; 3 Abdominal Centre/Endocrinology, Helsinki University Hospital, Helsinki, Finland; 4 Folkhalsan Research Center, Helsinki, Finland; 5 Institute for Molecular Medicine Finland FIMM, University of Helsinki, Helsinki, Finland; 6 Lund University Diabetes Center, Malmo, Sweden

**Keywords:** Early-onset type 2 diabetes, Perinatal factors, Birth weight, Preterm birth, Fetal programming, Maternal diabetes

## Abstract

Incidence of early-onset (< 40 years) type 2 diabetes (T2D) is increasing. While multiple risk factors have been identified, particularly obesity and low socioeconomic status, early-life factors are hypothesised to play a role via fetal programming. We investigated sociodemographic and early-life factors in relation to early-onset T2D using a family-based design that accounts for shared genetic and environmental factors. We included 1,814,062 individuals born in Sweden 1983 to 2002 with follow-up data until 2020, and identified early-onset (age 19–39) T2D cases (n = 3505) through National Diabetes, Patient and Prescribed Drug Registers. Perinatal and sociodemographic factors were retrieved from registers. We used a cohort and sibling design, with multivariable-adjusted Cox proportional hazards regression. Sociodemographic factors associated with early-onset T2D included low parental education, single parenthood, younger parental age and non-Swedish origin. The latter association did not remain after mutual adjustment. Regarding perinatal factors, a higher incidence was noted in relation to lower birth weight (hazard ratio 2.38 [95% confidence interval: 1.98–2.87] and 1.43[1.33–1.54] for < 2500 g and 2500-3500 g, respectively, vs 3500-4500 g), small-for-gestational-age (SGA) (2.24[1.96–2.56]), large-for-gestational-age (LGA) (1.19[1.01–1.39]), and maternal obesity (2.34[2.04–2.69]), diabetes (1.59[1.36–1.85]), smoking (1.59[1.48–1.71]), and infection (1.21[1.03–1.41]) during pregnancy. In the sibling analysis, only low birth weight and SGA remained associated with early-onset T2D. Early-onset T2D is associated with sociodemographic and multiple perinatal factors; only growth restriction likely reflects fetal programming, while other perinatal-related associations might involve confounders. This study highlights the need for early-life, targeted strategies to prevent T2D and reduce health inequities.

**Supplementary Information:**

The online version contains supplementary material available at 10.1007/s10654-025-01261-6.

## Background

Type 2 diabetes (T2D) has been consistently associated with early-life conditions, particularly low birth weight (LBW, considered an indicator of adverse uterine conditions interacting with fetal growth), preterm birth, and maternal obesity and diabetes during pregnancy [1, 2]. Albeit also criticized, the Barker hypothesis—also referred to as the fetal programming hypothesis—posits that adverse exposures during early life, such as maternal metabolic disorders, trigger fetal adaptive mechanisms that can lead to adverse metabolic outcomes later in life [3]. This hypothesis is supported by the consistent associations observed between perinatal factors and metabolic diseases, including T2D, across different cohorts worldwide, as well as experimental evidence from animal models [1, 2]. However, the proposed pathophysiological mechanisms, including epigenetic modifications, alterations in body composition, and pancreatic beta-cell dysfunction, are diverse and not yet fully elucidated [1].

Although primarily affecting middle-aged or elderly adults, T2D is increasing among young people (< 40 years) [4]. This group, termed “early-onset T2D”, has seen a global increase in prevalence, rising from 2.9% to 3.8% between 2013 and 2021 in the adults aged 20–39 years [5]. This is concerning considering that T2D seems to be a more aggressive disease when diagnosed early, carrying higher risks of complications, comorbidities, and death compared to T2D diagnosed at later ages [5, 6]. Like T2D at higher ages, early-onset T2D is closely linked to obesity, family history of diabetes, low socioeconomic status and certain ethnic backgrounds [7]. It is however conceivable that the pathways to obesity and subsequent T2D are different in early-onset T2D, primarily involving exposures accumulated in early life rather than in adulthood. Fetal programming may thus play an even more significant role for the development of early-onset T2D.

Studies on youth-onset (< 19 years) T2D provide support for the hypothesis of a strong influence of fetal programming, noting high risks associated with intrauterine exposure to maternal diabetes and obesity [8–10]. As an example, the SEARCH study estimated odds ratios of youth-onset T2D at 5.7 for gestational diabetes and 2.8 for maternal obesity [8]. Regarding T2D in young adults, a limited number of studies indicate increased risks related to being first born, LBW, being small-for-gestational-age (SGA) and preterm birth [11–13]. However, a comprehensive analysis of the risk of early-onset T2D in relation to a wide range of early-life factors has not yet been conducted.

Furthermore, it is challenging to separate the effects of fetal programming from those of genetic or environmental factors linked to maternal conditions and the long-term risk of diabetes in the offspring. Family-based designs can alleviate such confounding, e.g. by comparing siblings with different perinatal exposures. Thereby, the influence of fetal exposures on diabetes risk can be assessed while partly accounting for genetic susceptibility and lifestyle factors shared between siblings like infant feeding, childhood diet, physical activity, and family sociodemographic characteristics [14].

This study aimed to investigate the incidence of early-onset T2D in relation to sociodemographic and perinatal factors – including maternal obesity, diabetes, smoking or infection during pregnancy, pre-eclampsia, birth order, gestational age, birth weight, size for gestational age and mode of delivery – in the entire Swedish population born since 1983, using information from a wide range of high-quality registers. To reduce confounding, we complemented a traditional cohort design with a family-based design, comparing the incidence of early-onset T2D among siblings born to the same mother with different perinatal exposures[15]. This quasi-experimental design is made possible by the world’s largest national multigenerational register [16, 17].

## Methods

### Study population

We used personal identification numbers (PIN) to connect data from several Swedish registers, including the National Medical Birth Register (MBR), the Swedish Multi-Generation Register (MGR), the National Diabetes Register (NDR), the National Patient Register (NPR), the National Prescribed Drug Register (NPDR), the Health Insurance and Labour Market Studies register (LISA), as well as the Swedish emigration and death registers [16, 18–22].

MBR, which has been created in 1973, compiles standardized data from perinatal exams for all births in Sweden, including the international classification of diseases (ICD) codes for certain maternal diagnoses [18]. Our study included all individuals born in Sweden and recorded in the MBR from 1983 to 2002 (1,960,785 live births). These years were selected because some early-life exposures were not documented in the MBR before 1983 (specifically maternal body mass index [BMI], maternal smoking, and family situation at childbirth), and because we set the starting age of follow-up to be 18 years (data availability for identification of diabetes cases ends in 2020). This implies that we could follow people to a maximum of age 37 (people born 1983). Thus, we focused on early-onset T2D (≤ 37 years) and excluded youth-onset cases (< 18 years) to minimize the risk of including T1D cases since previous studies indicated very low rates of youth-onset T2D in Sweden [13, 23].

We excluded pregnancies with multiple births (50,066, 2.6%) and participants without linkages to the MGR or with system-identified duplicated or reused PINs (160, < 1%). Additionally, participants whose mothers were not identified in the MGR or had a duplicated or reused PIN were excluded (1518, < 1%). Participants that could not be linked to their fathers were retained in the study, considering paternal information missing.

**Fig. 1:**
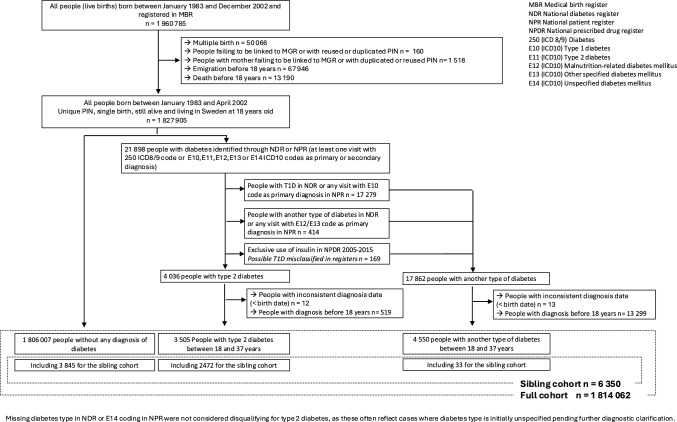
Population selection

The exclusion of participants who emigrated from Sweden or died before reaching 18 years of age (81,136, 4%) and those diagnosed with any type of diabetes before age 18 or without a reliable diagnosis date (13,843, < 1%) resulted in a final cohort of 1,814,062 individuals. For the sibling cohort, we included only sibling groups (born from the same mother) with at least one sibling affected by T2D (n = 6350). The population selection is summarized in Fig. 1. The study was approved by the Swedish Ethics Review Board (registration number: Dnr 2021–02881).

### Information on diabetes

We identified diabetes cases using available data from the NDR and the NPR until 2020. Additionally, we used the NPDR to enhance precision regarding diabetes type and onset date. The NDR, established in 1996, encompasses both primary and secondary diabetes care in Sweden, and was estimated to account for 87% of all individuals living with diabetes in the country in 2019 [24]. The NPR includes inpatient (hospital discharge) and outpatient specialist care data since 1964 and 2001, respectively [20].The NPDR, starting from 2005, records all prescribed medications dispensed in Sweden, categorized according to the Anatomical Therapeutic Chemical (ATC) classification [21].

We defined diabetes cases (n = 21,898) as participants with at least one record in the NDR or at least one visit documented in the NPR with a primary or secondary diagnosis code of “250” (ICD-8/ICD-9 code) or “E10-E14” (ICD-10 codes).

Cases were classified as T2D (n = 4036) if they had no record of another type of diabetes in NDR or NPR (primary diagnosis). To preserve sensitivity, missingness on diabetes type in NDR or E14 coding (“unspecified diabetes mellitus”) in NPR were not considered disqualifying conditions for T2D as these situations can occur when diabetes type is unspecified at early stage after diagnosis and additional diagnostic information is needed, especially in young adults. To ensure specificity, subjects with other types of diabetes formally recorded in NDR or NPR (i.e., E10, E12, E13) and individuals exclusively treated with insulin, for the whole follow-up period, according to data available from NPDR, were censored at the age of diagnosis (n = 17,862). Age of diagnosis was based on the first record in any of the registers.

### Familial and sociodemographic factors

We determined parental age (< 20 years, ≥ 20 & < 25 years, ≥ 25& < 30 years and ≥ 30 year) and family situation (two-parent, single-parent, or other situation) at birth using data from the MBR, and the highest parental educational level at birth (categorized as “Primary” for up to compulsory education [≤ 16 years old], “Secondary” for upper secondary education [16–19 years old], and “University” for college/university and further education) from the LISA register [22]. Additionally, the parental countries of birth were identified via the total population register and parents were categorized as Sweden or non-Sweden born, distinctly for the mother and the father. Finally, we identified parental diabetes cases (regardless of type) using the same registers as for study participants. For maternal history of diabetes, we additionally accounted for the information available in the MBR regarding pregestational and gestational diabetes diagnoses using ICD codes (ICD-8: 250; ICD-9: 250, 648A, 648W; ICD-10: O240-44) and self-report. Then, we combined all the information to identify the lifetime maternal and paternal history of diabetes. To examine maternal diabetes as a perinatal risk factor, we separately assess the T2D incidence in relation to maternal diabetes with a diagnosis before the participant’s birth (thus accounting for pregestational and gestational diabetes) from cases with a diagnosis after the participant’s birth.

### Pre- and perinatal factors

Using data from the MBR, we examined maternal BMI (< 18.5 kg/m^2^[underweight], 18.5–25 kg/m^2^[normal], 25–30 kg/m^2^ [overweight], and ≥ 30 kg/m^2^ [obesity]), and maternal smoking (none, 1 to 9 cigarettes per day, and ≥ 10 cigarettes per day) at the time of enrollment in maternal health care (first trimester). Additional factors studied included the mode of delivery (C-section or vaginal), gestational age (categorized as extremely to very preterm [≥ 22 to < 32 weeks], moderate to late preterm [≥ 32 to < 37 weeks], early term [≥ 37 to < 39 weeks], full term [≥ 39 weeks]), size for gestational age (small [SGA] or large [LGA] for gestational age, defined as birth weight below -2 or above + 2 standard deviations for the sex-specific distribution of birth weight in people born at the same gestational age), birth weight (< 2500 g, ≥ 2500 g and < 3500 g, ≥ 3500 g and < 4500 g, ≥ 4500 g), and birth order [25, 26].

Additionally, we identified pre-eclampsia using ICD codes (ICD-8: 637; ICD-9: 642E-G; ICD-10: O14-O15) from the MBR, and maternal infections during pregnancy using infection-related ICD codes from the NPR, as detailed in supplementary Table S1.

### Statistical analyses

We described the distribution of exposures and covariates using frequencies and proportions for categorical variables, and means and standard deviations (SD) or median and interquartile range (IQR) for continuous variables. We used Cox proportional hazards (PH) regression models to estimate the hazard ratios (HRs) and 95% confidence intervals (CIs) for the incidence of early-onset T2D in relation to early-life exposures. The follow-up period was calculated from the participants’ 18th birthday to the occurrence of diabetes, death, emigration, or April 21, 2020. The models used age as the time scale.

Missing values for categorical variables were treated as a separate category. For continuous covariates, the median value of the distribution was imputed for individuals with missing values, with an additional variable to indicate if the value was imputed or not. Overall, the percentage of missing values was low (ranging from 0 to 6%) except for maternal BMI (29%), which is due to the gradual introduction of the variable in the 1980s, as well as a complete lack of data for two consecutive years caused by an error in the data collection system rather than selective reporting of the mothers [18].

In the full cohort analysis, Cox models were conducted with a cluster-robust standard error to account for familial clustering among siblings born to the same mother. Each association between exposure and early-onset T2D was separately adjusted for sex and year of birth (Model 1). Then, all perinatal and familial factors were mutually adjusted for (Model 2) and finally (Model 3) we added adjustment for parental lifetime history of diabetes.

In the sibling analysis, we performed the Cox model with stratification on sibling groups for suitable exposures (sex, maternal BMI, diabetes, smoking and infection during pregnancy, pre-eclampsia, gestational age, birth weight, birth size, and mode of delivery), excluding exposures that were quasi-systematically concordant (family socio-educational background and parental lifetime history of diabetes) or systematically discordant (year of birth, birth order and parental age at childbirth) within siblings. Each association between exposure and early-onset T2D was separately adjusted for sex (Model 1). Then, all perinatal factors were mutually adjusted for (Model 2). Methods and interpretation of the sibling analysis are further detailed in the supplementary material.

Finally, we tested for an interaction between sex and perinatal exposures associated with early-onset T2D in the full and sibling cohort analyses, as sex-specific susceptibility to the intrauterine environment has previously been described, and sex differences have been observed in attributable risk factors for early-onset T2D [4]. To do so, we compared models with and without an interaction term, using the Likelihood ratio test.

We verified the PH assumption by plotting Schoenfeld residuals and performed stratified analysis if a violation was observed. All analyses were conducted using R, version 4.3.1.

## Results

### Incidence of early-onset T2D

In the full cohort, we identified 3,505 cases of early-onset T2D over 17,449,085 person-years of follow-up, resulting in an incidence rate of 20.1 per 100 000 person-years, 20.8 in men and 19.3 in women. The median (IQR) duration of follow-up was 9.8 (8.8) years and the median age at diagnosis of T2D was 27.0 (7.4) years. Overall, 89% of the participants were born from a two-parents family, and 39% had at least one parent with a college or university-level of education. Eighty-one percent of the participants had both parents born in Sweden, 11% of fathers had diabetes vs 6% of mothers. Preterm birth accounted for 5% of the cohort and mean (SD) birth weight was 3,542 (553) g. The sibling cohort involved 64,040 person-years and 2,472 cases of early-onset T2D. The characteristics of the participants in the full and sibling cohorts, according to early-onset T2D status, are detailed in Table 1.

**Table 1:** Characteristics of participants by early-onset type 2 diabetes (T2D) status in full and sibling cohorts

	Full cohort				Sibling cohort			
	No T2D		T2D		No T2D		T2D	
n	1 810 557		3 505		3878		2472	
Men n (%)	929,921	(51.4)	1872	(53.4)	2016	(52.0)	1298	(52.5)
Year of birth n (%)								
1983 to 1987	449,962	(24.9)	1832	(52.3)	1141	(29.4)	1156	(46.8)
1988 to 1992	545,854	(30.1)	1176	(33.6)	1508	(38.9)	940	(38.0)
1993 to 1997	466,730	(25.8)	428	(12.2)	849	(21.9)	333	(13.5)
1998 to 2002	348,011	(19.2)	69	(2.0)	380	(9.8)	43	(1.7)
Family situation n (%)								
Two-parent family	1,607,780	(88.8)	2977	(84.9)	3363	(86.7)	2107	(85.2)
Single mother or another situation	89,280	(4.9)	307	(8.8)	274	(7.1)	202	(8.2)
Missing	113,497	(6.3)	221	(6.3)	241	(6.2)	163	(6.6)
Highest parental degree at the child’s birth n (%)								
≤ Compulsory education	134,200	(7.4)	571	(16.3)	618	(15.9)	369	(14.9)
Upper secondary	971,159	(53.6)	2258	(64.4)	2567	(66.2)	1641	(66.4)
College/university	704,308	(38.9)	675	(19.3)	692	(17.8)	462	(18.7)
Missing	890	(0.0)	1	(0.0)	1	(0.0)	0	(0.0)
Parental country of birth n (%)								
Both parents born in Sweden	1,467,386	(81.0)	2723	(77.7)	3060	(78.9)	1962	(79.4)
Sweden-born mother only	100,903	(5.6)	235	(6.7)	245	(6.3)	157	(6.4)
Sweden-born father only	86,267	(4.8)	180	(5.1)	175	(4.5)	114	(4.6)
Both parents born outside Sweden	146,496	(8.1)	331	(9.4)	370	(9.5)	218	(8.8)
Missing	9505	(0.5)	36	(1.0)	28	(0.7)	21	(0.8)
Lifetime history of diabetes in the mother n (%)								
History of diabetes	110,984	(6.1)	1089	(31.1)	1032	(26.6)	705	(28.5)
Lifetime history of diabetes in the father n (%)								
History of diabetes	188,909	(10.4)	1232	(35.1)	1268	(32.7)	882	(35.7)
Missing data	9505	(0.5)	36	(1.0)	28	(0.7)	21	(0.8)
Age of the mother n (%)								
< 20 years	45,128	(2.5)	199	(5.7)	130	(3.4)	158	(6.4)
≥ 20 to < 25 years	366,061	(20.2)	1007	(28.7)	913	(23.5)	821	(33.2)
≥ 25 to < 30 years	663,730	(36.7)	1180	(33.7)	1376	(35.5)	896	(36.2)
≥ 30 years	735,638	(40.6)	1119	(31.9)	1459	(37.6)	597	(24.2)
Age of the mother (mean (SD))	29.00	(5.08)	27.74	(5.42)	28.55	(5.22)	26.74	(4.89)
Age of the father n (%)								
< 20 years	11,217	(0.6)	54	(1.5)	26	(0.7)	35	(1.4)
≥ 20 to < 25 years	183,255	(10.1)	535	(15.3)	496	(12.8)	436	(17.6)
≥ 25 to < 30 years	542,994	(30.0)	1087	(31.0)	1072	(27.6)	853	(34.5)
≥ 30 years	1,063,586	(58.7)	1793	(51.2)	2256	(58.2)	1127	(45.6)
Missing	9505	(0.5)	36	(1.0)	28	(0.7)	21	(0.8)
Age of the father (mean (SD))	31.88	(6.01)	31.03	(6.41)	31.87	(6.36)	30.15	(5.97)

	Full cohort				Sibling cohort			
	No type 2 diabetes		Type 2 diabetes		No type 2 diabetes		Type 2 diabetes	
n	1 810 557		3 505		3878		2472	
BMI of the mother in early pregnancy n (%)								
< 18.5 kg/m2	66,109	(3.7)	107	(3.1)	107	(2.8)	85	(3.4)
≥ 18.5 to < 25 kg/m2	911,149	(50.3)	1402	(40.0)	1328	(34.2)	986	(39.9)
≥ 25 to < 30 kg/m2	235,361	(13.0)	557	(15.9)	715	(18.4)	413	(16.7)
≥ 30 kg/m2	74,748	(4.1)	276	(7.9)	437	(11.3)	180	(7.3)
Missing	523,190	(28.9)	1163	(33.2)	1291	(33.3)	808	(32.7)
BMI of the mother in early pregnancy (mean (SD))	22.96	(3.36)	23.76	(3.97)	24.44	(4.26)	23.71	(3.82)
Exposure to maternal diabetes during pregnancy n (%)	22,876	(1.3)	211	(6.0)	190	(4.9)	119	(4.8)
Smoking during pregnancy n (%)								
No smoking	1,328,290	(73.4)	1813	(51.7)	2173	(56.0)	1328	(53.7)
1 to 9 cigarettes per day	233,893	(12.9)	746	(21.3)	752	(19.4)	500	(20.2)
10 or more cigarettes per day	138,043	(7.6)	690	(19.7)	713	(18.4)	465	(18.8)
Missing	110,331	(6.1)	256	(7.3)	240	(6.2)	179	(7.2)
Infection during pregnancy n (%)	61,399	(3.4)	163	(4.7)	167	(4.3)	102	(4.1)
Pre-eclampsia or eclampsia n (%)	50,573	(2.8)	163	(4.7)	117	(3.0)	102	(4.1)
Birth order n (%)								
1	748,863	(41.4)	1487	(42.4)	748	(19.3)	1077	(43.6)
2	651,182	(36.0)	1104	(31.5)	1321	(34.1)	818	(33.1)
≥ 3	410,512	(22.7)	914	(26.1)	1809	(46.6)	577	(23.3)
Birth order (mean (SD))	1.92	(1.03)	1.97	(1.11)	2.62	(1.33)	1.92	(1.08)
Gestational age n (%)								
Extremely to very preterm (≥ 22 to < 32 weeks)	9806	(0.5)	51	(1.5)	23	(0.6)	33	(1.3)
Moderate to late preterm (≥ 32 to < 37 weeks)	79,866	(4.4)	255	(7.3)	218	(5.6)	162	(6.6)
Early birth term (≥ 37 to < 39 weeks)	333,863	(18.4)	728	(20.8)	815	(21.0)	511	(20.7)
Full term (≥ 39 weeks)	1,384,253	(76.5)	2457	(70.1)	2812	(72.5)	1759	(71.2)
Missing	2769	(0.2)	14	(0.4)	10	(0.3)	7	(0.3)
Birth weight n (%)								
< 2500 g	57,209	(3.2)	264	(7.5)	143	(3.7)	173	(7.0)
≥ 2500 g and < 3500 g	760,840	(42.0)	1733	(49.4)	1648	(42.5)	1232	(49.8)
≥ 3500 g and < 4500 g	920,690	(50.9)	1367	(39.0)	1843	(47.5)	966	(39.1)
≥ 4500 g	65,657	(3.6)	124	(3.5)	237	(6.1)	91	(3.7)
Missing	6161	(0.3)	17	(0.5)	7	(0.2)	10	(0.4)
Birth weight (g) (mean (SD))	3543	(552)	3369	(654)	3548	(611)	3376	(644)
Size for gestational age n (%)								
Small for gestational age	45,381	(2.5)	245	(7.0)	132	(3.4)	171	(6.9)
Normal for gestational age	1,694,157	(93.6)	3058	(87.2)	3481	(89.8)	2162	(87.5)
Large for gestational age	62,261	(3.4)	172	(4.9)	248	(6.4)	123	(5.0)
Missing	8758	(0.5)	30	(0.9)	17	(0.4)	16	(0.6)
Mode of delivery n (%)								
Vaginal delivery	1,603,729	(88.6)	3017	(86.1)	3467	(89.4)	2169	(87.7)
C section	206,828	(11.4)	488	(13.9)	411	(10.6)	303	(12.3)
Censored during follow-up n (%)								
Other type of diabetes	4550	(0.3)			33	(0.9)		
Emigration during follow-up (> 18 years old)	51,398	(2.8)			83	(2.1)		
Death during follow-up (> 18 years old)	66	(0.0)			0	(0.0)		

### Sociodemographic factors, parental diabetes and incidence of early-onset T2D

The incidence of early-onset T2D was higher in men than in women (hazard ratio [HR] 1.12, 95% confidence interval [CI]: 1.05–1.20) and increased with year of birth, being 60% higher in those born in 1998–2002 compared with 1983–1987 (Table 2, model 3). Having a mother or a father or both parents born outside Sweden vs. two Sweden-born parents was associated with a higher risk (+ 35%, + 19% and + 58%, respectively) of early-onset T2D (Table 2, model 1). The incidence was also higher in those born to a single mother (1.83[1.62–2.06]), in those whose parents had low education level (3.54[3.16—3.96] for compulsory-only education vs college-to-university education) or were young at childbirth (2.29[1.97–2.67] for mother age < 20 vs ≥ 30, and 2.49[1.90–3.26] for father age < 20 vs ≥ 30) and in those with parental lifetime history of diabetes (5.61[5.21–6.04] when in the mother, 3.75[3.49–4.03] when in the father). These associations were attenuated but remained after being mutually adjusted (Table 2, model 3) except for the association with parental country of birth which was attenuated almost to the null after adjustment for parental educational level and diabetes.

**Table 2:** Incidence of early-onset type 2 diabetes and hazard ratio (CI95) according to exposure variables in the full cohort

	Cases	Person-years	Incidence rate*	Model 1	Model 2	Model 3
Sex						
Women	1 633	8 457 221	19.31		1.00	1.00
Men	1 872	8 991 864	20.82		1.12 (1.04—1.19)	1.12 (1.05—1.20)
Year of birth						
1983 to 1987	1 832	7 235 448	25.32		1.00	1.00
1988 to 1992	1 176	6 250 068	18.82		1.05 (0.96—1.14)	1.19 (1.09—1.30)
1993 to 1997	428	3 228 202	13.26		1.00 (0.88—1.13)	1.30 (1.15—1.47)
1998 to 2002	69	735 366	9.38		1.10 (0.85—1.42)	1.60 (1.23—2.07)
Family situation						
Two-parent family	2 977	15 538 042	19.16	1.00	1.00	1.00
Single mother or another situation	307	875 154	35.08	1.83 (1.62—2.06)	1.24 (1.10—1.41)	1.21 (1.07—1.37)
Highest parental degree at the child’s birth						
Up to compulsory education	571	1 459 330	39.13	3.54 (3.16—3.96)	2.14 (1.89—2.42)	1.81 (1.59—2.05)
Upper secondary	2 258	9 518 494	23.72	2.26 (2.07—2.46)	1.71 (1.56—1.88)	1.54 (1.41—1.69)
College/university	675	6 464 983	10.44	1.00	1.00	1.00
Parental country of birth						
Both parents born in Sweden	2 723	14 399 265	18.91	1.00	1.00	1.00
Sweden-born mother only	235	940 577	24.98	1.35 (1.18—1.55)	1.19 (1.04—1.37)	1.14 (1.00—1.31)
Sweden-born father only	180	812 620	22.15	1.19 (1.02—1.38)	1.17 (1.00—1.36)	1.09 (0.94—1.27)
Both parents born outside Sweden	331	1 206 038	27.45	1.58 (1.41—1.78)	1.33 (1.18—1.50)	0.99 (0.88—1.12)
Lifetime diabetes history in the mother						
No diabetes	2 416	16 213 281	14.90	1.00		1.00
History of diabetes	1 089	1 235 779	88.12	5.61 (5.21—6.04)		4.02 (3.68—4.38)
Lifetime diabetes history in the father						
No diabetes	2 237	15 242 367	14.68	1.00		1.00
History of diabetes	1 232	2 116 135	58.22	3.75 (3.49—4.03)		3.16 (2.94—3.41)
Age of the mother at the child’s birth						
< 20 years	199	482 875	41.21	2.29 (1.97—2.67)	1.67 (1.38—2.03)	1.84 (1.51—2.23)
≥ 20 to < 25 years	1 007	3 883 381	25.93	1.46 (1.34—1.59)	1.32 (1.17—1.49)	1.44 (1.28—1.63)
≥ 25 to < 30 years	1 180	6 468 308	18.24	1.06 (0.97—1.15)	1.10 (1.00—1.20)	1.18 (1.08—1.30)
≥ 30 years	1 119	6 614 521	16.92	1.00	1.00	1.00
Age of the father at the child’s birth						
< 20 years	54	115 027	46.95	2.49 (1.90—3.26)	1.22 (0.90—1.66)	1.47 (1.09—1.99)
≥ 20 to < 25 years	535	1 958 707	27.31	1.45 (1.31—1.59)	0.99 (0.88—1.13)	1.15 (1.01—1.30)
≥ 25 to < 30 years	1 087	5 426 169	20.03	1.08 (1.00—1.17)	0.97 (0.89—1.06)	1.07 (0.98—1.17)
≥ 30 years	1 793	9 858 598	18.19	1.00	1.00	1.00
Maternal BMI in early pregnancy						
< 18.5 kg/m2	107	774 751	13.81	0.79 (0.65—0.96)	0.65 (0.53—0.79)	0.68 (0.56—0.83)
≥ 18.5 and < 25 kg/m2	1 402	8 746 670	16.03	1.00	1.00	1.00
≥ 25 and < 30 kg/m2	557	1 813 525	30.71	2.16 (1.96—2.39)	1.99 (1.80—2.20)	1.54 (1.39—1.70)
≥ 30 kg/m2	276	477 259	57.83	4.60 (4.04—5.24)	3.67 (3.21—4.19)	2.34 (2.04—2.69)
Maternal diabetes during pregnancy						
No	3 294	17 245 135	19.10	1.00	1.00	1.00
Yes	211	203 924	103.47	5.69 (4.95—6.56)	4.79 (4.15—5.53)	1.59 (1.36—1.85)
Maternal smoking during pregnancy						
No smoking	1 813	12 194 972	14.87	1.00	1.00	1.00
Smoking	1 436	4 153 779	34.57	2.18 (2.03—2.34)	1.71 (1.58—1.84)	1.59 (1.48—1.71)
1 to 9 cigarettes per day	746	2 570 517	29.02	1.84 (1.69—2.01)	1.51 (1.38—1.65)	1.43 (1.31—1.56)
10 or more cigarettes per day	690	1 583 261	43.58	2.73 (2.50—2.99)	2.01 (1.83—2.21)	1.83 (1.67—2.02)
Infection during pregnancy						
No	3 342	16 900 556	19.77	1.00	1.00	1.00
Yes	163	548 529	29.72	1.50 (1.28—1.75)	1.24 (1.06—1.46)	1.21 (1.03—1.41)
Pre-eclampsia						
No	3 342	16 961 609	19.70	1.00	1.00	1.00
Yes	163	487 477	33.44	1.65 (1.41—1.93)	1.26 (1.07—1.49)	1.14 (0.96—1.34)
Birth order						
1	1 487	7 162 640	20.76	1.00	1.00	1.00
2	1 104	6 241 830	17.69	0.85 (0.79—0.92)	0.97 (0.89—1.06)	0.96 (0.88—1.04)
≥ 3	914	4 044 616	22.60	1.08 (1.00—1.18)	1.13 (1.02—1.25)	1.04 (0.94—1.15)
Gestational age						
Non-full-term birth (< 39 weeks)	1 034	4 155 016	24.89	1.32 (1.23—1.42)	0.96 (0.88—1.05)	0.94 (0.86—1.02)
Extremely to very preterm (≥ 22 to < 32 weeks)	51	91 662	55.64	3.03 (2.30—4.00)	1.17 (0.84—1.63)	1.15 (0.83—1.60)
Moderate to late preterm (≥ 32 to < 37 weeks)	255	790 902	32.24	1.70 (1.50—1.94)	0.95 (0.81—1.13)	0.93 (0.79—1.09)
Early birth term (≥ 37 to < 39 weeks)	728	3 272 452	22.25	1.18 (1.09—1.28)	0.96 (0.88—1.05)	0.94 (0.86—1.02)
Full term (≥ 39 weeks)	2 457	13 264 589	18.52	1.00	1.00	1.00
Birth weight						
< 2500 g	264	567 773	46.50	2.95 (2.58—3.37)	2.40 (1.99—2.90)	2.38 (1.98—2.87)
≥ 2500 g and < 3500 g	1 733	7 484 730	23.15	1.48 (1.37—1.59)	1.42 (1.32—1.53)	1.43 (1.33—1.54)
≥ 3500 g and < 4500 g	1 367	8 742 745	15.64	1.00	1.00	1.00
≥ 4500 g	124	589 109	21.05	1.36 (1.13—1.63)	1.23 (1.02—1.47)	1.13 (0.94—1.36)
Size for gestational age**						
Small for gestational age	245	461 832	53.05	2.78 (2.44—3.17)	2.31 (2.02—2.64)	2.24 (1.96—2.56)
Normal for gestational age	3 058	16 321 465	18.74	1.00	1.00	1.00
Large for gestational age	172	572 920	30.02	1.62 (1.39—1.90)	1.32 (1.13—1.55)	1.19 (1.01—1.39)
Mode of delivery						
Vaginal delivery	3 017	15 555 373	19.40	1.00	1.00	1.00
C section	488	1 893 712	25.77	1.33 (1.21—1.47)	1.02 (0.92—1.13)	0.99 (0.89—1.09)

*For 100 000 person years

Model 1: each association with early-onset T2D is separately adjusted for sex and year of birth

Model 2: mutual adjustment for sex, year of birth, parental country of birth, parental highest educational level, family situation, parental age at delivery, maternal BMI, diabetes, smoking and infection during pregnancy, pre-eclampsia, birth order, gestational age, birth weight, and mode of delivery

Model 3: Model 2 + adjustment for parental lifetime history of diabetes

**In Model 2 and 3, size for gestational age was adjusted for the same variables except gestational age and birth weight

We detected violations to the PH assumption for some exposures (sex, birth year and parental history of diabetes) and consequently stratified the analyses by attained age (Supplemental Table S2). These analyses indicate a reverse association between sex and early-onset T2D, as the incidence was higher in women compared to men (1.29[1.15–1.44]) before 25 years, and higher in men compared to women (1.40[1.28–1.52]) after 25 years. Additionally, the risk associated with year of birth and parental history of diabetes was higher before 25 years.

### Perinatal factors and incidence of early-onset T2D

In the full cohort, we found a dose–response association between gestational age and early-onset T2D, with the highest HR (3.03[2.30–4.00]) for extremely to very preterm birth (< 32 weeks vs ≥ 39 weeks) (Table 2, model 1). We found a U-shaped association between birth weight and early-onset T2D with the lowest incidence observed in participants born with birth weight between 3500 and 4500 g. In comparison, participants born < 2500 g and 2500 to 3500 g had a higher risk of early-onset T2D (2.95[2.58–3.37] and 1.48[1.37–1.59], respectively), as well as those born ≥ 4500 g (1.36[1.13–1.63]). The association with size for gestational age showed the same pattern, with a higher risk for small (2.78 [2.44–3.17]) and large (1.62 [1.39–1.90]) compared to normal for gestational age. After mutual adjustment (Table 2, model 2 and 3), the higher risk observed for LBW and SGA was slightly attenuated, while the other associations were markedly attenuated. These results were consistent with those in the sibling cohort (Table 3), where we observed a higher risk of early-onset T2D only for LBW (2.48[1.65–3.72] and 1.35[1.15–1.58] for < 2500 g and 2500 to 3500 g, respectively, vs 3500 g to 4500 g) and SGA (2.05[1.47–2.86]).

**Table 3:** Incidence of early-onset type 2 diabetes and hazard ratios (CI95) according to exposure variables in the sibling cohort

	Cases	Person-years	Incidence rate*	Model 1	Model 2
Sex					
Women	1 174	29 757	39.45		1.00
Men	1 298	34 283	37.86		0.90 (0.80—1.02)
BMI of the mother in early pregnancy					
< 18.5 kg/m2	85	2 224	38.22	1.08 (0.72–1.63)	1.04 (0.69–1.57)
≥ 18.5 and < 25 kg/m2	986	25 355	38.89	1.00	1.00
≥ 25 and < 30 kg/m2	413	10 599	38.97	0.94 (0.76–1.16)	0.94 (0.76–1.17)
≥ 30 kg/m2	180	4 553	39.53	0.87 (0.63–1.18)	0.92 (0.67–1.27)
Exposure to maternal diabetes during pregnancy					
No	2 353	61 465	38.28	1.00	1.00
Yes	119	2 575	46.21	1.18 (0.76–1.85)	1.25 (0.79–1.96)
Smoking during pregnancy					
No smoking	1 328	34 513	38.48	1.00	1.00
Smoking	965	25 198	38.30	1.08 (0.84–1.39)	1.02 (0.79–1.32)
1 to 9 cigarettes per day	500	13 202	37.87	1.04 (0.80–1.36)	0.99 (0.75–1.29)
10 or more cigarettes per day	465	11 996	38.76	1.15 (0.86–1.53)	1.08 (0.80–1.45)
Infection during pregnancy					
No	2 370	61 417	38.59	1.00	1.00
Yes	102	2 623	38.89	0.98 (0.72–1.34)	0.94 (0.68–1.29)
Pre-eclampsia					
No	2 370	61 800	38.35	1.00	1.00
Yes	102	2 240	45.53	1.18 (0.80–1.73)	1.08 (0.73–1.61)
Gestational age					
Non-full-term birth (< 39 weeks)	706	17 581	40.16	1.14 (0.97–1.33)	0.97 (0.82–1.15)
Extremely to very preterm (≥ 22 to < 32 weeks)	33	503	65.60	4.30 (1.68–10.96)	2.25 (0.83–6.07)
Moderate to late preterm (≥ 32 to < 37 weeks)	162	3 690	43.90	1.21 (0.90–1.63)	0.87 (0.63–1.22)
Early birth term (≥ 37 to < 39 weeks)	511	13 388	38.17	1.09 (0.92–1.29)	0.98 (0.82–1.16)
Full term (≥ 39 weeks)	1 759	46 298	37.99	1.00	1.00
Birth weight					
< 2500 g	173	3 127	55.33	2.60 (1.83–3.69)	2.48 (1.65–3.72)
≥ 2500 g and < 3500 g	1 232	30 139	40.88	1.34 (1.15–1.56)	1.35 (1.15–1.58)
≥ 3500 g and < 4500 g	966	27 643	34.94	1.00	1.00
≥ 4500 g	91	2 945	30.90	0.73 (0.51–1.03)	0.72 (0.51–1.03)
Size for gestational age**					
Small for gestational age	171	3 048	56.10	2.11 (1.51–2.93)	2.05 (1.47–2.86)
Normal for gestational age	2 162	57 393	37.67	1.00	1.00
Large for gestational age	123	3 260	37.73	1.01 (0.74–1.40)	1.02 (0.73–1.40)
Mode of delivery					
Vaginal delivery	2 169	57 054	38.02	1.00	1.00
C section	303	6 986	43.37	1.19 (0.89–1.60)	1.05 (0.77–1.44)

*For 1 000 person years

Model 1: each association with early-onset T2D is separately adjusted for sex

Model 2: mutual adjustment for sex, maternal BMI, diabetes, smoking and infection during pregnancy, pre-eclampsia, gestational age, birth weight, and mode of delivery

**In Model 2, size for gestational age was adjusted for the same variables except gestational age and birth weight

Regarding other perinatal exposures, we observed a higher incidence (Table 2, model 1) of early-onset T2D in participants exposed to maternal obesity (4.60 [4.04–5.24]), diabetes (5.69 [4.95–6.56]), smoking (2.18 [2.03–2.34]) or infection (1.50 [1.28–1.75]) during pregnancy, as well as pre-eclampsia (1.65 [1.41–1.93]) and C-Sect. (1.33 [1.21–1.47]). These associations were attenuated but remained significant after mutual adjustment (except for pre-eclampsia and C-section) (Table 2). However, all these associations were null in the sibling analysis (Table 3). Notably, exposure to maternal obesity (0.92 [0.67 – 1.27]) and diabetes (1.25 [0.79—1.96]) did not alter the risk of early-onset T2D among siblings born to the same mother.

The PH assumption was respected for all perinatal exposures except for the associations with maternal obesity and diabetes, which showed attenuation during follow-up (Supplementary Table S2 and S3 present results stratified by age at onset in the full cohort and the sibling cohort, respectively).

**Fig. 2:**
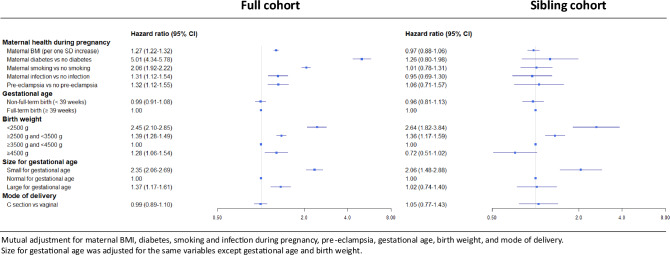
Forest plot summarizing the associations between early life exposures and early-onset type 2 diabetes in the full cohort and in the sibling cohort. Results are mutually adjusted for all variables presented in the forest plot except for the size for gestational age. Size for gestational age was adjusted for all variables except gestational age and birth weight

Figure 2 presents a summary of the full cohort and sibling cohort analyses, both based on mutual adjustment for the same set of variables to ensure comparability of estimates between the two cohorts.

## Discussion

This study is the first to provide a comprehensive analysis of the incidence of T2D in young adults in relation to early-life exposures based on European nationwide data. In a national cohort involving the whole Swedish population, we observed that low and high birth weight (also when corrected for gestational age), and exposure to maternal obesity, diabetes, smoking, or infection during pregnancy were associated to early-onset T2D. However, in a subset cohort that included T2D cases and their T2D-free siblings as controls (sibling cohort), we could only confirm the higher risk for people born with LBW or SGA. These findings support the hypothesis of a fetal programming of early-onset T2D in the context of growth restriction, whereas associations with other perinatal factors, notably exposure to maternal diabetes and obesity, may reflect genetic susceptibility or the influence of environmental factors shared among family members.

Growth restriction resulting in LBW has been associated with long-term adverse metabolic outcomes, including T2D [1, 12]. Although there are arguments for causality based on animal studies, human data are observational. Therefore, providing a quasi-experimental design through a large sibling analysis raises the level of evidence. In terms of pathophysiology, several mechanisms have been proposed, some of which have been validated in experimental models. One key hypothesis involves rapid postnatal catch-up growth, typically observed following intrauterine growth restriction. It has been linked to unfavorable changes in body composition, including altered fat distribution with increased visceral adiposity [27]. These changes are known to negatively affect insulin sensitivity, further exacerbating the risk of glucose dysregulation. In addition, growth restriction has been associated with impaired pancreatic beta cell development, which can result in a lifelong reduction in insulin secretory capacity [28]. More recently, epigenetic modifications, such as DNA methylation changes in genes involved in metabolic regulation, and alterations in the early-life gut microbiome have also been proposed as contributing factors to long-term health adverse outcomes [29, 30]. In any case, history of fetal growth restriction should have clinical implications in diabetes screening strategy. The American Diabetes Association has previously identified SGA as a risk factor that should prompt diabetes screening in asymptomatic children and adolescents who are overweight or obese [31]. The results of our study are consistent with this statement and even support extending this recommendation to young adults.

On the other hand, our findings challenge the fetal programming hypothesis of early-onset T2D regarding in utero exposure to maternal obesity and diabetes. We observed an association between prenatal exposure to maternal obesity or diabetes and early-onset T2D in participants of the total cohort, aligning with previous findings [8–10]. But the association turned out to be neutral in the sibling cohort. Therefore, the association observed in the total cohort is likely reflecting genetic susceptibility for diabetes, and/or environmental factors shared among family members such as diet and physical inactivity. Indeed, studies that compared T2D within siblings exposed and unexposed to maternal diabetes during pregnancy are scarce and relates to specific populations. Notably, one study investigating T2D in a Native American population (aged 6 to 24 years), showed a significant excess risk of T2D in siblings exposed to maternal diabetes during pregnancy [32]. Thus, it is possible that maternal diabetes affects fetal programming of children and adolescent-onset T2D but not in older ages, or that this effect varies depending on ethnicity. To our knowledge, no similar sibling study has been conducted in a European population with early-T2D as the outcome.

Regarding other perinatal exposures, we found an association between high birth weight, preterm birth (although non-persistent in fully adjusted models) and early-onset T2D in the total cohort, aligning with previous literature [12, 13]. In addition, we found an association with maternal smoking, maternal infection and early-onset T2D. To our knowledge, these last associations have not been identified previously. However, all these associations were null in the sibling cohort, and are, as for the association with maternal diabetes and obesity, likely to result from confounders.

Parental history of diabetes was the strongest risk factor for early-onset T2D in our study. The associations were most pronounced for T2D diagnosed before age 25. This is consistent with the previous observation of an inverse association between the number of T2D-affected family members and the age of diabetes onset [33]. Previous studies have linked early-onset T2D to lower socioeconomic status, parental educational level and younger age at childbirth [7, 11]. In confirmation hereof, we observed a higher risk of early-onset T2D in people born from non-Sweden-born parents, young parents, parents with low level of education, and single-parent families. These associations may reflect more early-life exposure to unhealthy dietary habits and less physical activity, contributing to obesity which in turn, is closely tied to disparities in educational level and socio-economic status as well as the risk of T2D. Consequently, significant public health improvements could likely be achieved by developing targeted prevention strategies for high-risk families, aiming to reduce the risk of obesity and related adverse metabolic outcomes, particularly in young adults with a history of LBW or SGA.

Finally, this study confirms that early-onset T2D should be considered a growing threat to public health as we observed an increasing risk of early-onset T2D along with the year of birth, especially in people younger than 25 years, which is consistent with the increasing incidence of early-onset T2D observed worldwide [4]. Using a strict definition of T2D (excluding people with conflicting diagnoses or treatments), which prioritizes specificity over sensitivity, we have for the first time provided a reliable estimate of the incidence of early-onset T2D in the Swedish population. We also observed a time-dependent association between sex and early-onset T2D, with an excess risk for women before 25 years of age and an excess risk for men thereafter. This pattern has been observed in several cohort studies of early-onset T2D and is thought to be related to sex-specific weight gain and distribution before and after puberty [5]. Indeed, young women are more affected by high BMI, which is the most important attributable risk factor for early-onset T2D [4]. However, BMI at the time of T2D onset was not available in this study, limiting our ability to directly test this hypothesis. Additionally, the possibility of a detection bias cannot be ruled out, as women are more likely to seek healthcare earlier, particularly in relation to reproductive health.

This study has many strengths, including the collection of national data from reliable registries with multiple variables allowing for the consideration of a wide range of exposures and confounders while reducing selection and avoiding recall biases. In particular, the sibling analysis, using the larger national multigenerational registry in the world, allowed us to investigate the fetal programming hypothesis of early-onset T2D in relation to in utero exposure to maternal diabetes, which, to our knowledge, has never been done in a European nationwide cohort [16, 17]. However, this study has several limitations. Because early-onset T2D cases could not be clinically validated, we have excluded individuals younger than 18 years to avoid misclassification with type 1 diabetes, resulting in lack of data on childhood and adolescent-onset T2D. Regarding the incidence of T2D in individuals aged 18–40, it is likely to be underestimated due to undiagnosed T2D cases or management in primary care centers whose data are captured (with high coverage) in NDR but not in NPR. Another limitation relates to the assessment of exposures, as data collection is based on national registries. Thus, some exposures were not considered at all, particularly nutritional habits, since unavailable in national registries. Additionally, some studied exposures may be underestimated, such as minor maternal infections that do not require specialist care. Overall, misclassification on exposures or T2D diagnosis might have biased some associations toward the null, which is further concerning for the sibling cohort that has a limited sample size and for whom more statistical power is necessary to detect weak associations. Finally, the external validity of the study may be limited to European populations. Conducting similar analyses in other populations, particularly those with a high prevalence of early-onset type 2 diabetes, would be of considerable interest.

## Conclusions

To conclude, this study highlights the growing incidence of early-onset T2D and emphasizes the urgent need for prevention and screening strategies that go beyond traditional risk factors. Given the strong link between fetal growth restriction and later-life T2D, tailored interventions must incorporate early-life health conditions, particularly in vulnerable populations. Addressing this issue is not only a medical priority but also a socioeconomic imperative, as disadvantaged communities often face higher exposure to T2D risk factors, exacerbating health inequities. Proactive measures can reduce the long-term socio-economic burden of diabetes while improving outcomes for at-risk groups.

## Supplementary Information

Below is the link to the electronic supplementary material.

Supplementary file1 (PDF 118 KB)

## Data Availability

Data used for the study is register-based data that is pseudonymised and thus subject to General Data Protection Regulation (GDPR) and cannot be shared openly. Only metadata is published openly. Underlying data from the registers can be made available upon request to the Research Data Office at the Karolinska Institutet via rdo@ki.se after ensuring compliance with relevant legislation and GDPR.

## Abbreviations

BMI: Body mass index
ICD: International classification of diseases
LBW: Low birth weight
LGA: Large for gestational age
LISA: Health Insurance and Labour Market Studies register [*Longitudinell Integrationsdatabas för Sjukförsäkrings- och Arbetsmarknadsstudier]*
MBR: Medical birth register
MGR: Multi-Generation Register
NDR: National diabetes register
NPDR: National prescribed drugs register
NPR: National patient resgister
PIN: Personal identification number
SGA: Small for gestational age
T2D: Type 2 diabetes
