# Supplementary material for: Fetal programming of early-onset type 2 diabetes: a Swedish nationwide cohort and sibling analysis
Source: Eur J Epidemiol. 2025 Jun 24;40(7):845–57. doi: 10.1007/s10654-025-01261-6 (PMC12304039; doi:10.1007/s10654-025-01261-6)
Supplement: Supplementary file 1 — Supplementary file1 (PDF 118 KB) [file 10654_2025_1261_MOESM1_ESM.pdf]

Supplemental text. Methods related to the sibling comparison design

The sibling comparison cohort (n=6,350) includes all sibling groups (with the same mother) where at least one sibling had early-onset T2D. Only sibling groups discordant for perinatal exposure contribute to the risk estimates for the exposure. However, sibling groups concordant for perinatal exposure are informative for the risk estimates of covariates and are thus included in the analysis. Within this cohort, we compared the hazard of early-onset T2D in exposed individuals to their unexposed siblings. This sibling comparison design can be seen as a nested case-control design where each case of early-onset T2D ("index case") is matched with their T2D-free siblings by age. This means each index case is compared for exposure status to all their T2D-free siblings alive at the index case's age of diabetes onset. This approach inherently controls for unmeasured confounders shared between siblings, including genetic and environmental factors. An estimate from this sibling comparison design approaching 1 suggests that the association identified in the full cohort analysis may be influenced by confounding factors shared among siblings.

Table S1. ICD codes used from the national patient register to identify infection during pregnancy.

	ICD-10	ICD-9	ICD-8
Any infection	A00-A99; B00-B89; B95-B99; E06.0; G00-G02; G04; G05-G08; H00; H03; H10; H66; H70; I00-I02; I30; I33; J00-J06; J09; J10-J18; J20-J22; J32; J36; J85; J86; K35-K37; K57.0; K57.2; K57.4; K57.8; K61; K63.0; K67; K75.0; K77.0; K81.0; K83.0; K85; L00-L04; L08; M00; M01; M46.3; M86; N10-N12; N30; N61; N70-N76; O07.0; O07.5; O08.0; O23; O35.3; O41.1; O75.3; O98	001-018; 020-027; 030-041; 045-057; 060-066; 070-088; 090-104; 110-112; 114-118; 120-136; 245A; 254B; 320; 321; 323; 324; 372A; 372C; 382; 383; 390; 391; 392; 420; 421; 460-466; 473; 475; 480-487; 510; 513; 540-542; 566; 572A; 575A; 590; 595; 614-616; 646F; 646G; 647; 658E; 659C; 659D; 680-684; 686; 730	000–018; 020-027; 030-043; 045; 046; 050-057; 060-065; 067; 068; 070-076; 078-104; 110-117; 120-134; 136; 320-322; 360,00; 360,01; 361; 362; 381-383; 390-392; 420-422; 460-466; 470-474; 480-486; 490; 501; 503; 510; 513; 540; 541; 562,02; 562,11; 566; 567; 569,00; 569,01; 569,02; 569,03; 569,04; 577,01; 590; 595; 597; 612-614; 616; 620; 622; 630; 635; 636,00; 670-672; 678; 680-684; 686; 710; 720; 732; 761,00; 761,20; 761,30; 761,40; 761,80; 763,00; 999,30
Virus infection	A08; A60; A80-A89; A92-A99; B00-B09; B15-B19; B20-B34; B97; G02.0; G05.1; J00; J09-J12; O35.3; O98.4-98.5	045-066; 070-072; 074-075; 077; 079; 321E, H; 323A, C, D; 460; 647F, G; 771A	040-043; 045-065; 067-072; 074; 075; 078; 460; 470-474; 480; 761,20; 761,30
Bacterial infection	A00-A05; A15-A19; A20-A28; A65-A79; B95-B96; E06.0; G00; G01; G04.2; G05.0; I00-I02; J13-J15; J85; K57.0; K57.2; K57.4; K57.8; K61; K63.0; K81.0; K85; L00-L04; M00; N61; N74; O07.0; O07.5; O23; O41.1; O75.3; O98.0-98.2	001-005; 010-041; 073; 076; 080-083; 087-098; 100-104; 245A; 320; 390-392; 481; 482; 513; 566; 646F, G; 647A, B, D; 658E; 659D; 681-684	000-005; 010-018; 020-039; 073; 076; 080-083; 088-104; 320,00-320,80; 361; 390-392; 481; 482; 513; 562,02; 562,11; 566; 569,00; 577,01; 630; 635; 636,00; 678,02; 680-684; 710; 761,00; 763,00; 999,30
Genitourinary infection	A50-A64; O23; N10-N12; N30; N70-N76	016; 090-099; 590; 595; 614-616; 646G	016; 090-099; 590; 595; 612-614; 620; 622; 635
Respiratory infection	A15-A16; A37-A38; B27; B39-42; B44; B59; J00-J06; J09-J18; J20-J22; J32; J36; J85; J86	010-012; 033-034; 460-466; 473; 475; 480-487; 510; 513	010-012; 460-466; 470-473; 480-486; 501; 503; 510; 513
Gastrointestinal Infection	A00-A09	001-009	000-009
Other ICD codes (considered having lower accuracy level)	E32; H01; H04; H05; H13; H16; H32; H44; H60; H62; I40; J35; J39; K04; K05; K11; K12; K14; K65; L05; L30; M49; M60; M63; M65; N13; N15; N34; N39; N98; T80-T84; T88	279; 360; 370; 373; 375; 376; 380; 422; 478; 511; 522; 523; 527-529; 562; 567; 597; 599; 611; 685; 711; 728; 790; 996; 998; 999	366-369; 380; 384; 508; 511; 522; 527; 528; 542; 599; 611; 629; 789; 998

Table S2. Hazard ratios (CI95) according to the period of follow-up in the full cohort.

	From 18 to 25 years old	From 25 to 37 years old
Sex		
Women	1.29 (1.15 - 1.44)	1.00
Men	1.00	1.40 (1.28 - 1.52)
Year of birth		
1983 to 1992	1.00	1.00
1993 to 2002	1.30 (1.14 - 1.47)	0.85 (0.63 - 1.15)
Family situation		
Two-parent family	1.00	1.00
Single mother or another situation	0.94 (0.75 - 1.18)	1.38 (1.19 - 1.61)
Highest parental degree at the child's birth		
Up to compulsory education	1.72 (1.39 - 2.12)	1.87 (1.60 - 2.18)
Upper secondary	1.47 (1.26 - 1.70)	1.61 (1.43 - 1.81)
College/university	1.00	1.00
Parental country of birth n (%)		
Both parents born in Sweden	1.00	1.00
Sweden-born mother only	1.44 (1.17 - 1.75)	0.98 (0.81 - 1.17)
Sweden-born father only	1.17 (0.92 - 1.50)	1.04 (0.86 - 1.27)
Both parents born outside Sweden	1.00 (0.82 - 1.21)	0.99 (0.85 - 1.16)
Lifetime diabetes history in the mother		
No diabetes	1.00	1.00
History of diabetes	3.97 (3.44 - 4.59)	3.99 (3.59 - 4.43)
Lifetime diabetes history in the father		
No diabetes	1.00	1.00
History of diabetes	3.62 (3.20 - 4.09)	2.90 (2.64 - 3.18)
Age of the mother at the child's birth		
<20 years	2.02 (1.45 - 2.80)	1.74 (1.37 - 2.21)
≥ 20 to <25 years	1.40 (1.15 - 1.70)	1.47 (1.26 - 1.70)
≥ 25 to <30 years	1.17 (1.01 - 1.35)	1.19 (1.06 - 1.34)
≥ 30 years	1.00	1.00
Age of the father at the child's birth		
<20 years	1.50 (0.90 - 2.50)	1.46 (1.00 - 2.12)
≥ 20 to <25 years	1.11 (0.89 - 1.38)	1.17 (1.00 - 1.36)
≥ 25 to <30 years	1.04 (0.90 - 1.20)	1.09 (0.97 - 1.22)
≥ 30 years	1.00	1.00
BMI of the mother in early pregnancy		
<18.5 kg/m2	0.95 (0.68 - 1.33)	0.58 (0.45 - 0.74)
≥ 18.5 and <25 kg/m2	1.00	1.00
≥ 25 and <30 kg/m2	1.89 (1.60 - 2.22)	1.38 (1.21 - 1.58)
≥ 30 kg/m2	2.81 (2.30 - 3.43)	2.08 (1.72 - 2.53)
Exposure to maternal diabetes during pregnancy		
No	1.00	
Yes	1.92 (1.54 - 2.38)	1.33 (1.07 - 1.65)
Smoking during pregnancy		
No smoking	1.00	1.00
1 to 9 cigarettes per day	1.54 (1.33 - 1.78)	1.35 (1.21 - 1.51)
10 or more cigarettes per day	1.85 (1.57 - 2.17)	1.79 (1.60 - 2.01)
Infection during pregnancy		
No	1.00	1.00

Yes	1.25 (0.97 - 1.61)	1.17 (0.95 - 1.43)
Pre-eclampsia		
No	1.00	1.00
Yes	1.07 (0.81 - 1.41)	1.17 (0.95 - 1.43)
Birth order		
1	1.00	1.00
2	1.00 (0.88 - 1.15)	0.93 (0.83 - 1.03)
≥ 3	1.00 (0.85 - 1.17)	1.07 (0.94 - 1.21)
Gestational age		
Extremely to very preterm (≥ 22 to < 32 weeks)	0.97 (0.54 - 1.75)	1.23 (0.83 - 1.83)
Moderate to late preterm (≥ 32 to < 37 weeks)	1.07 (0.81 - 1.40)	0.85 (0.69 - 1.04)
Early birth term (≥ 37 to < 39 weeks)	1.09 (0.94 - 1.25)	0.85 (0.76 - 0.95)
Full term (≥ 39 weeks)	1.00	1.00
Birth weight n (%)		
<2500 g	1.97 (1.44 - 2.68)	2.68 (2.13 - 3.37)
≥2500 g and <3500 g	1.22 (1.08 - 1.39)	1.57 (1.43 - 1.73)
≥3500 g and <4500 g	1.00	1.00
≥4500 g	1.33 (1.02 - 1.74)	0.99 (0.77 - 1.28)
Size gestational age** n (%)		
Small for gestational age (< 2 SD)	2.24 (1.79 - 2.82)	2.23 (1.89 - 2.64)
Normal for gestational age	1.00	1.00
Large for gestational age (>2 SD)	1.46 (1.17 - 1.83)	0.99 (0.79 - 1.24)
Mode of delivery		
Vaginal delivery	1.00	1.00
C section	1.04 (0.88 - 1.22)	0.95 (0.83 - 1.09)

Mutual adjustment for sex, year of birth, parental country of birth, parental highest educational level, family situation, parental age at delivery, maternal BMI, diabetes, smoking and infection during pregnancy, pre-eclampsia, birth order, gestational age, birth weight, mode of delivery and parental lifetime history of diabetes.

**Size for gestational age was adjusted for the same variables except gestational age and birth weight.

Table S3. Hazard ratios (CI95) according to the period of follow-up in the sibling cohort.

	From 18 to 25 years old	From 25 to 37 years old
Sex		
Women	1.54 (1.29 - 1.84)	1.00
Men	1.00	1.29 (1.07 - 1.54)
BMI of the mother in early pregnancy		
< 18.5 kg/m2	1.14 (0.63 - 2.06)	0.98 (0.55 - 1.76)
≥ 18.5 and < 25 kg/m2	1.00	1.00
≥ 25 and < 30 kg/m2	1.10 (0.81 - 1.49)	0.82 (0.59 - 1.12)
≥ 30 kg/m2	1.08 (0.71 - 1.64)	0.75 (0.44 - 1.26)
Exposure to maternal diabetes during pregnancy		
No	1.00	1.00
Yes	1.49 (0.83 - 2.70)	0.80 (0.38 - 1.68)
Smoking during pregnancy		
No smoking	1.00	1.00
1 to 9 cigarettes per day	1.19 (0.81 - 1.74)	0.84 (0.56 - 1.26)
10 or more cigarettes per day	1.18 (0.78 - 1.78)	1.04 (0.67 - 1.61)
Infection during pregnancy		
No	1.00	1.00
Yes	0.90 (0.58 - 1.38)	0.96 (0.59 - 1.56)
Pre-eclampsia		
No	1.00	1.00
Yes	1.13 (0.63 - 2.03)	0.99 (0.57 - 1.72)
Gestational age		
Extremely to very preterm (≥ 22 to < 32 weeks)	1.42 (0.32 - 6.28)	2.91 (0.72 - 11.72)
Moderate to late preterm (≥ 32 to < 37 weeks)	0.88 (0.56 - 1.39)	0.85 (0.52 - 1.39)
Early birth term (≥ 37 to < 39 weeks)	1.15 (0.89 - 1.47)	0.82 (0.64 - 1.05)
Full term (≥ 39 weeks)	1.00	1.00
Birth weight		
<2500 g	2.60 (1.49 - 4.55)	2.29 (1.23 - 4.25)
≥2500 g and <3500 g	1.30 (1.04 - 1.63)	1.50 (1.19 - 1.88)
≥3500 g and <4500 g	1.00	1.00
≥4500 g	0.99 (0.63 - 1.55)	0.40 (0.22 - 0.75)
Size for gestational age*		
Small for gestational age	2.11 (1.31 - 3.41)	1.93 (1.20 - 3.10)
Normal for gestational age	1.00	1.00
Large for gestational age	1.10 (0.72 - 1.67)	0.86 (0.51 - 1.47)
Mode of delivery		
Vaginal delivery	1.00	1.00
C section	1.17 (0.76 - 1.78)	0.92 (0.57 - 1. 48)

Mutual adjustment for sex, maternal BMI, diabetes, smoking and infection during pregnancy, pre-eclampsia, gestational age, birth weight, and mode of delivery.

*Size for gestational age was adjusted for the same variables except gestational age and birth weight.
